# 70. Changes in Invasive Pneumococcal Disease among Adults Living with HIV Following Introduction of 13-Valent Pneumococcal Conjugate Vaccine, 2008–2018

**DOI:** 10.1093/ofid/ofab466.070

**Published:** 2021-12-04

**Authors:** Almea Matanock, Jianmin Li, William Adih, Wei Xing, William Schaffner, Nisha B Alden, Lee Harrison, Susan Petit, Joan Baumbach, Arthur Reingold, Olivia Almendares, Ryan Gierke, Corinne Holtzman, Monica M Farley, Ann Thomas, Tamara Pilishvili, Miwako Kobayashi

**Affiliations:** 1 CDC, Atlanta, Georgia; 2 Centers for Disease Control and Prevention, Atlanta, Georgia; 3 Weems Design Studio Inc. Contractor to CDC, Atlanta, Georgia; 4 Vanderbilt University Medical Center, Nashville, Tennessee; 5 Colorado Department of Public Health and Environment, Denver, Colorado; 6 University of Pittsburgh Medical Center, Pittsburgh, Pennsylvania; 7 Connecticut Department of Public Health, Hartford, Connecticut; 8 New Mexico Departmet of Health, Santa Fe, New Mexico; 9 UC Berkeley, Berkeley, California; 10 Minnesota Department of Health, St. Paul, Minnesota; 11 Emory University, Atlanta, Georgia; 12 Oregon Public Health Division, Portland, Oregon; 13 Centers for Disease Control and Prevention, Atlanta, GA, USA, Atlanta, Georgia

## Abstract

**Background:**

People living with HIV (PLHIV) are at increased risk of invasive pneumococcal disease (IPD). The 13-valent pneumococcal conjugate vaccine (PCV13) was recommended for children in 2010, and for immunocompromised adults (including PLHIV) in series with 23-valent polysaccharide vaccine (PPSV23) in 2012. We evaluated changes in IPD incidence in adults ≥19 years old by HIV status after PCV13 introduction and proportion of remaining IPD due to serotypes included in the 15- (PCV15) and 20-valent (PCV20) conjugate vaccines expected to be licensed in 2021.

**Methods:**

IPD cases were identified through CDC’s Active Bacterial Core surveillance (ABCs). HIV status was obtained from medical records. Isolates were serotyped by Quellung reaction, or whole-genome sequencing and grouped into PCV13-types, PPV11-types (unique to PPSV23), or non-vaccine types. We estimated IPD incidence (cases per 100,000 people) using national projections of ABCs cases as numerators and national case-based HIV surveillance (PLHIV) or US census data (non-PLHIV) as denominators. We compared IPD incidence in 2011–12 and 2017–18 to pre-PCV13 baseline (2008–09) by serotype groups. We assessed the proportion of IPD due to serotypes included in PCV15 and PCV20.

**Results:**

Overall IPD incidence at baseline was 306.7 for PLHIV and 15.2 for non-PLHIV. From baseline to 2017–18, IPD incidence declined in PLHIV (-40.3%; 95% CI: -47.7, -32.3%) and non-PLHIV (-28.2%; 95% CI: -30.9, -25.5%). The largest reductions were in PCV13-type IPD during both periods (-44.2% for PLHIV and -42.2% for non-PLHIV in 2011–12; -72.5% for PLHIV and -62.2% for non-PLHIV in 2017–18) compared to baseline (Figures 1, 2). In 2017–2018, overall IPD and PCV13-type rates were 16.8 (95% CI: 15.1, 18.5) and 12.6 (95% CI: 9.9, 15.3) times as high in PLHIV vs non-PLHIV, respectively; PCV13, PCV15/non-PCV13, and PCV20/non-PCV15 serotypes comprised 21.5%, 11.2% and 16.5% of IPD in PLHIV.

IPD incidence rates among adults aged ≥19 years old by serotype group in PLHIV, 2008–2018

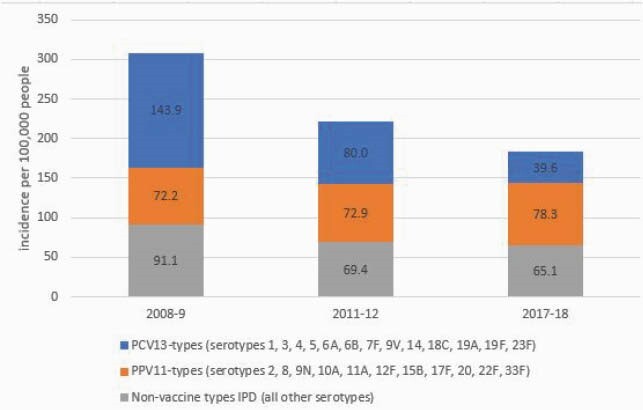

IPD incidence rates among adults aged ≥19 years old by serotype group in non-PLHIV, 2008–2018

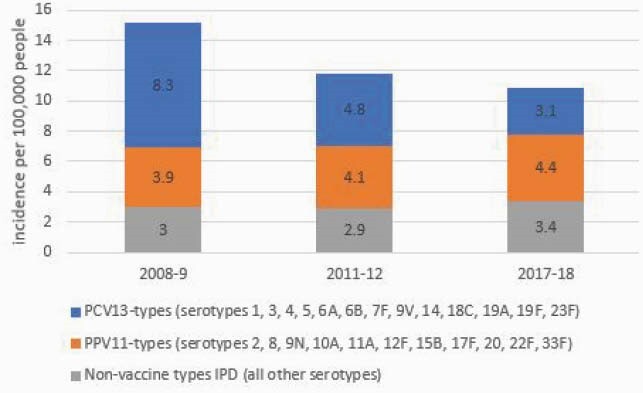

**Conclusion:**

IPD rates declined significantly in both PLHIV and non-PLHIV during the study period due to reductions in PCV13-type IPD; however, IPD rates remained 17-fold higher in PLHIV compared to non-PLHIV, mainly due to non-PCV13 types. Higher-valent pneumococcal conjugate vaccines provide opportunities to reduce some of the remaining IPD burden in PLHIV.

**Disclosures:**

**William Schaffner, MD**, **VBI Vaccines** (Consultant) **Lee Harrison, MD**, **GSK, Merck, Pfizer, Sanofi Pasteur** (Consultant)

